# Development of an intelligent decision support system for ischemic stroke risk assessment in a population-based electronic health record database

**DOI:** 10.1371/journal.pone.0213007

**Published:** 2019-03-13

**Authors:** Chen-Ying Hung, Ching-Heng Lin, Tsuo-Hung Lan, Giia-Sheun Peng, Chi-Chun Lee

**Affiliations:** 1 Department of Electrical Engineering, National Tsing Hua University, Hsinchu, Taiwan; 2 Department of Internal Medicine, Taipei Veterans General Hospital, Hsinchu Branch, Hsinchu, Taiwan; 3 Department of Nutrition, Hungkuang University, Taichung, Taiwan; 4 Department of Medical Research, Taichung Veterans General Hospital, Taichung, Taiwan; 5 Department of Healthcare Management, National Taipei University of Nursing and Health Sciences, Taipei, Taiwan; 6 MOST Joint Research Center for AI Technology and All Vista Healthcare, Taipei, Taiwan; Duke-NUS Medical School, SINGAPORE

## Abstract

**Background:**

Intelligent decision support systems (IDSS) have been applied to tasks of disease management. Deep neural networks (DNNs) are artificial intelligent techniques to achieve high modeling power. The application of DNNs to large-scale data for estimating stroke risk needs to be assessed and validated. This study aims to apply a DNN for deriving a stroke predictive model using a big electronic health record database.

**Methods and results:**

The Taiwan National Health Insurance Research Database was used to conduct a retrospective population-based study. The database was divided into one development dataset for model training (~70% of total patients for training and ~10% for parameter tuning) and two testing datasets (each ~10%). A total of 11,192,916 claim records from 840,487 patients were used. The primary outcome was defined as any ischemic stroke in inpatient records within 3 years after study enrollment. The DNN was evaluated using the area under the receiver operating characteristic curve (AUC or c-statistic). The development dataset included 672,214 patients (a total of 8,952,000 records) of whom 2,060 patients had stroke events. The mean age of the population was 35.5±20.2 years, with 48.5% men. The model achieved AUC values of 0.920 (95% confidence interval [CI], 0.908–0.932) in testing dataset 1 and 0.925 (95% CI, 0.914–0.937) in testing dataset 2. Under a high sensitivity operating point, the sensitivity and specificity were 92.5% and 79.8% for testing dataset 1; 91.8% and 79.9% for testing dataset 2. Under a high specificity operating point, the sensitivity and specificity were 80.3% and 87.5% for testing dataset 1; 83.7% and 87.5% for testing dataset 2. The DNN model maintained high predictability 5 years after being developed. The model achieved similar performance to other clinical risk assessment scores.

**Conclusions:**

Using a DNN algorithm on this large electronic health record database is capable of obtaining a high performing model for assessment of ischemic stroke risk. Further research is needed to determine whether such a DNN-based IDSS could lead to an improvement in clinical practice.

## Introduction

Globally, approximately 6.5 million stroke deaths happen each year–making stroke the second-leading cause of death and thus an important public health issue.[1] The mortality and disability associated with stroke significantly impact lives of patients and their families. Developing predictive risk assessment is essential in continuously improving stroke prevention by providing healthcare professionals reliable pre-screening analytics.[2,3] In fact, many existing clinical guidelines recommend the use of stroke risk assessment tools, e.g., the Framingham[4] and QRISK[5] scoring systems, to identify patients at a high risk of stroke.[6–8] However, large-scale deployment of these questionnaire-based assessments in outpatient departments or clinics is inefficient and impractical. This draw-back is especially evident when scaling up the assessment effort in places with large volumes of primary care, or for the general population. A scalable and reliable automated stroke risk assessment system could offer clinical decision support instruments for healthcare professionals and further benefit societal welfare.

Intelligent decision support systems (IDSS),[9–12] i.e., those developed based on artificial intelligence (AI) techniques (such as machine learning algorithms[13–15]), have demonstrated great achievement in a variety of clinical tasks in recent years.[16–18] In fact, as the volume of electronic data in healthcare system grows, these techniques have been successfully applied in disease identification and outcome prediction,[19–21] e.g., Parkinson's disease,[22] heart failure,[23] in-hospital mortality,[24] and coronary artery disease.[25,26] Among a wealth of machine learning methods, deep learning techniques have recently produced results surpassing the ability of trained human experts in tasks such as recognition of diabetic retinopathy[27–29] and melanoma skin lesions,[30] and detection of tumor metastases.[31] Deep learning is formulated as a mathematical neural network architecture consisting of multiple hidden layers with non-linear activation.[32] It is capable of modeling complex non-linear relationships between predictive variables without prior statistical assumptions.[33] Moreover, when given a sufficiently large amount of data, the DNN may outperform conventional statistical methods due to its non-linear learning structure.[15,32,34]

The electronic health record (EHR), by nature, is collected non-obtrusively in a large-scale long-term follow-up manner.[11] These properties along with the inclusion of diverse aspects of patients' health-related information make EHR a valuable data source for constructing automated risk assessment systems with deep learning techniques.[35] In fact, in our recent work, we have demonstrated that DNN can achieve a higher stroke occurrence predictive accuracy compared to other conventional machine learning methods when trained on the EHR database.[34] However, it is not known whether a DNN based IDSS would be more accurate than currently used clinical stroke risk assessment scores. Furthermore, the stability of the DNN model needs to be additionally validated across different time periods in order to fulfill real-world clinical practice requirements. The purpose of the present study is to investigate whether the DNN-based stroke predictive model derived from a large EHR database meets real world clinical practice requirements.

## Materials and methods

### Research database

The National Health Insurance program has been implemented in Taiwan since 1995 and covers more than 99% of the island’s population. The National Health Research Institute (NHRI) in Taiwan has established the database, National Health Insurance Research Database (NHIRD), from the claims data of the National Health Insurance program. We conducted a large population-based cohort study with a systematic sampling of patient data in the NHIRD. This random sample of patients (from January 1, 2000 to December 31, 2011 with a total of 1 million unique subjects) has been confirmed by the NHRI to be representative of the general population in Taiwan. The NHRI further made data available at the individual level in an anonymous format to protect the privacy of patients. The details of the NHIRD were described previously,[36] and this EHR database has been used for several important clinical studies.[37,38] The database can be accessed from the NHRI (https://nhird.nhri.org.tw/) or the Health and Welfare Data Science Center of Ministry of Health and Welfare, Taiwan. Ethics review was approved by the Institutional Review Board of Taichung Veterans General Hospital.

### Study population

In this study, we developed a predictive model to estimate 3 year risk of ischemic stroke in the general population. Patients aged 0 to 99 years who visited any outpatient departments or clinics between 1 January and 31 December in 2003 were identified. Patients were excluded if they had any pre-existing stroke records (International Classification of Diseases, Tenth Revision, Clinical Modification, [ICD-10-CM] code: I60~I69) at cohort entry. Following the exclusion process, our final dataset contained a total of 11,192,916 claim records from 840,487 patients. In order to develop and evaluate the DNN-based IDSS, these data were further assigned into one development dataset (including ~70% of total patients used for training algorithm, and ~10% for parameter tuning) and two testing datasets (each had ~10% of total patients). We utilized data from outpatient departments (within 3 years prior to the cohort entry) to generate predictive variables (features) and data from inpatient departments (within 3 years after the cohort entry) to retrieve target outcomes.

### Feature engineering and stroke event definition

In our previous study, we have established a feature engineering method to extract health-related information from the NHIRD database.[34,39] In brief, we gathered variables from outpatient database records within 3 years before study enrollment (containing information from 2000 to 2003); these variables included demographic data, healthcare costs and utilization, disease diagnoses, and medication use. Diagnostic records were re-classified by the first 3 characters of ICD-10-CM codes (for example, I10 for essential hypertension). While the original NHIRD database used ICD-9-CM codes to record diagnoses of diseases, we converted ICD-9-CM codes to ICD-10-CM codes according to the code-converting sheet provided by the Taiwan National Health Insurance Bureau. The records of medications were re-classified by the first 5 characters of ATC codes (for example, C10AA for statins). In order to additionally capture temporal information, we utilized the time stamp information in the process of variable computing (examples of the derived variables: mean and standard deviation [SD] of total insurance payments within 1 year before enrollment). Finally, we extracted a total of 7,932 predictive variables from the database.

**Fig 1:**
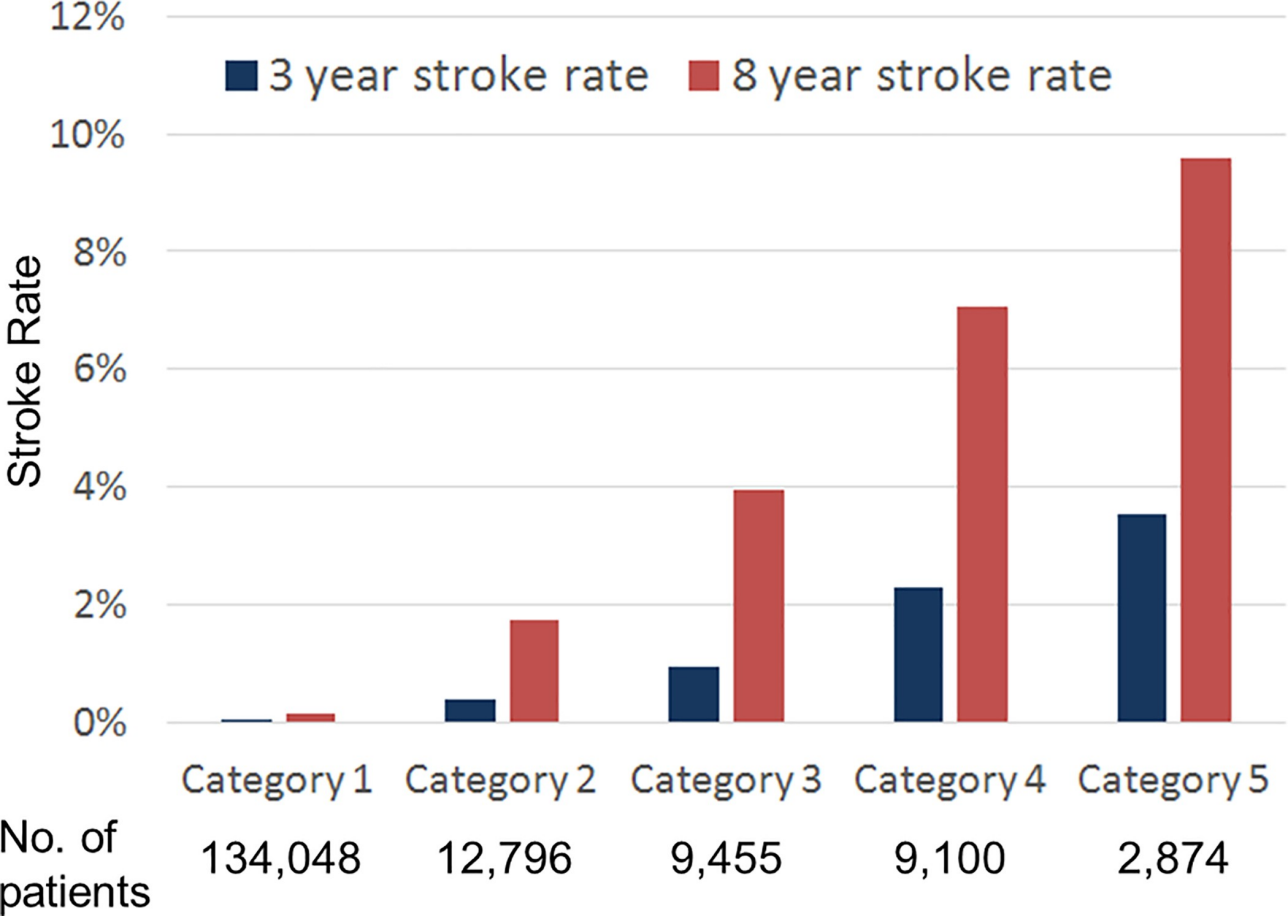
The 3 year and 8 year stroke rate of patients in the 5 risk categories in the testing datasets.

The primary outcome of this study was defined as any ischemic stroke (ICD-10-CM code: I63, equivalent to ICD-9-CM code: 433.01, 433.11, 433.21, 433.31, 433.81, 433.91, 434.01, 434.11, 434.91) recorded in the inpatient database within 3 years after patients being enrolled (from 2003 to 2006). This definition of ischemic stroke has been validated and suggested for NHIRD studies by Hsieh et al.[40] The positive predictive value and sensitivity for ischemic stroke detection were expected to be higher than 88% and 97% under this definition.[40] For further sensitivity analyses, we examined the developed algorithm with different outcome definitions (S1 Table). Extended 8-year outcome records were also retrieved from the database (from 2003 to 2011) for further stability testing of the predictive algorithm (see Fig 1).

### Development of the algorithm

The core mechanism of DNN is to train a multi-layered feedforward neural network to perform classifications.[32] The structure of our DNN model was composed of five fully connected layers, including an input layer, 3 hidden layers (each layer had 300 neurons), and an output layer (with only one neuron using a sigmoid function for binary stroke prediction). Hyperbolic tangent was used as the activation function and stochastic gradient descent was used as the optimization algorithm. We used part of the development dataset (~70% of total patients) to train the network weights using the method of back-propagation with cross entropy as the loss function.

In order to speed up the training process, we additionally utilized univariate Pearson correlation (a common data-distilled feature selection method[41,42] for selecting the most informative variables and reducing the number of input variables) to select the most relevant clinical factors from 7,932 variables and applied min-max normalization (rescaling every input variable to a range between 0 and 1) in order to train the neural network with numerical stability.[43] In the development of a machine learning or DNN model, selection of the best number of variables is usually an empirical decision, which depends on the purposes the model needs to achieve. Therefore, we performed experiments to determine the number of variables (S1 Fig). With more variables, performance of DNN models increased. However, there was only slight improvement (marginal benefit) after including more than 300 variables. Finally, we selected the most relevant 300 variables (S2 Table) for developing the stroke predictive model in this study. The use of 300 features represents a reasonable compromise between rapid computing and optimized prediction accuracy over time.

Because there are many more non-stroke cases than stroke cases, we randomly under-sampled the non-stroke cases in the development dataset in order to guarantee an almost identical class distribution between stroke and non-stroke cases. If no proper under-sampling is carried out, the training of a DNN model would converge to a solution categorizing every patient into the stroke class and ignoring the non-stroke class. Random under-sampling is often done to manage the class imbalance problem in data mining and machine learning.[44,45] Platt calibration (also known as Platt scaling), a method for better calibrating the probabilities of a machine learning model by fitting a logistic transformation to the model’s outputs, was applied for estimating stroke risk accurately.[46] Another part of the development dataset (~10% of total patients) was used to adjust the various hyper-parameters of the neural network (such as the early stopping criterion). The algorithm was implemented using the Keras (2015, GitHub) toolbox.

### Evaluations of the algorithm and sub-sampling experiments

Performance of the DNN stroke prediction model was examined on two testing datasets (each ~10% of total patients) based on person-level data. The metric used was the area under the receiver operating characteristic curve (AUC or c-statistic) values. The output layer of the DNN generates the probability of future stroke occurrence. Receiver operating characteristic curves were plotted by varying the operating threshold, i.e., that probability above which a patient is labeled “at risk of stroke”. Two operating cut points for the algorithm were selected from the development dataset. The high sensitivity operating cut point approximated a specificity of 80% and allowed a high sensitivity for disease screening use. The high specificity operating cut point corresponded to a sensitivity of 80% and allowed a high specificity for detecting high stroke risk patients that is suitable for preventive interventions. The receiver operating characteristic curve plots, AUC and 95% confidence intervals (CI) were computed using the Scikit-learn packages.[47] Model calibration was evaluated using calibration plots and the Hosmer-Lemeshow test.[48]

In order to assess whether the DNN model developed using data from 2003 would degrade over time, we tested the model at various time periods (2003, 2004, 2005, 2006, 2007 and 2008) on the two testing datasets. We also conducted sub-sampling experiments to understand the relationship between different amounts of training data and the model performance. The development dataset was divided into 8 separate sub-datasets (each included around 1 million records). We then iteratively added these sub-datasets into the DNN training process (S2 Fig). Performance of these models was also examined on the two testing datasets.

## Results

### Population characteristics

A total of 840,487 patients were enrolled in this study, of whom 672,214 were in the development dataset, 84,342 were in testing dataset 1 and 83,931 were in testing dataset 2. The development dataset included a total of 8,952,000 records. Testing dataset 1 and testing dataset 2 consisted of 1,118,320 records and 1,122,596 records, respectively. Patients’ demographics and characteristics of these datasets are summarized in Table 1. Each patient visited outpatient departments a median of 11 times (interquartile range, 5–20) in 2003. Within the 3 year period after enrollment, 2,060 patients in the development dataset had at least one stroke event. The mean age of the development dataset population was 35.5±20.2 years, with 48.5% men.

**Table 1:** Characteristics of development and testing datasets.

Characteristics	Development dataset		Testing dataset 1		Testing dataset 2	
No. of records	8,952,000		1,118,320		1,122,596	
No. of records with stroke events	43,911		4,578		5,000	
Patient demographics						
No. of patients	672,214		84,342		83,931	
No. of patients with stroke events	2,060		239		245	
No. of OPD visits in 2003, median (IQR)	11	(5–20)	11	(5–20)	11	(5–20)
Men, No. (%)	326,337	(48.5)	41,078	(48.7)	40,916	(48.7)
Age in years, mean (SD)	35.5	(20.2)	35.5	(20.2)	35.5	(20.3)
Co-morbidity, No. (%)						
Hypertension	79,696	(11.9)	9,968	(11.8)	9,944	(11.8)
Hyperlipidemia	50,929	(7.6)	6,395	(7.6)	6,288	(7.5)
Diabetes mellitus	38,635	(5.7)	5,017	(5.9)	4,780	(5.7)
Ischemic heart disease	32,126	(4.8)	3,975	(4.7)	3,973	(4.7)
Atrial fibrillation	1,958	(0.3)	235	(0.3)	224	(0.3)
Heart failure	7,959	(1.2)	989	(1.2)	1,020	(1.2)
Medication use, No. (%)						
Antiplatelet agents	75,252	(11.2)	9,425	(11.2)	9,311	(11.1)
Renin angiotensin system inhibitors	50,885	(7.6)	6,438	(7.6)	6,306	(7.5)
Beta blockers	87,051	(12.9)	11,019	(13.1)	10,966	(13.1)
Calcium channel blockers	67,373	(10.0)	8,438	(10.0)	8,268	(9.9)
Other antihypertensive drugs	70,876	(10.5)	9,023	(10.7)	8,844	(10.5)
Statins	18,567	(2.8)	2,382	(2.8)	2,312	(2.8)
Oral hypoglycemic agents	28,149	(4.2)	3,641	(4.3)	3,415	(4.1)
Insulins	4,746	(0.7)	637	(0.8)	592	(0.7)

OPD = outpatient department; IQR = interquartile range; SD = standard deviation.

### Performance of the algorithm

**Fig 2:**
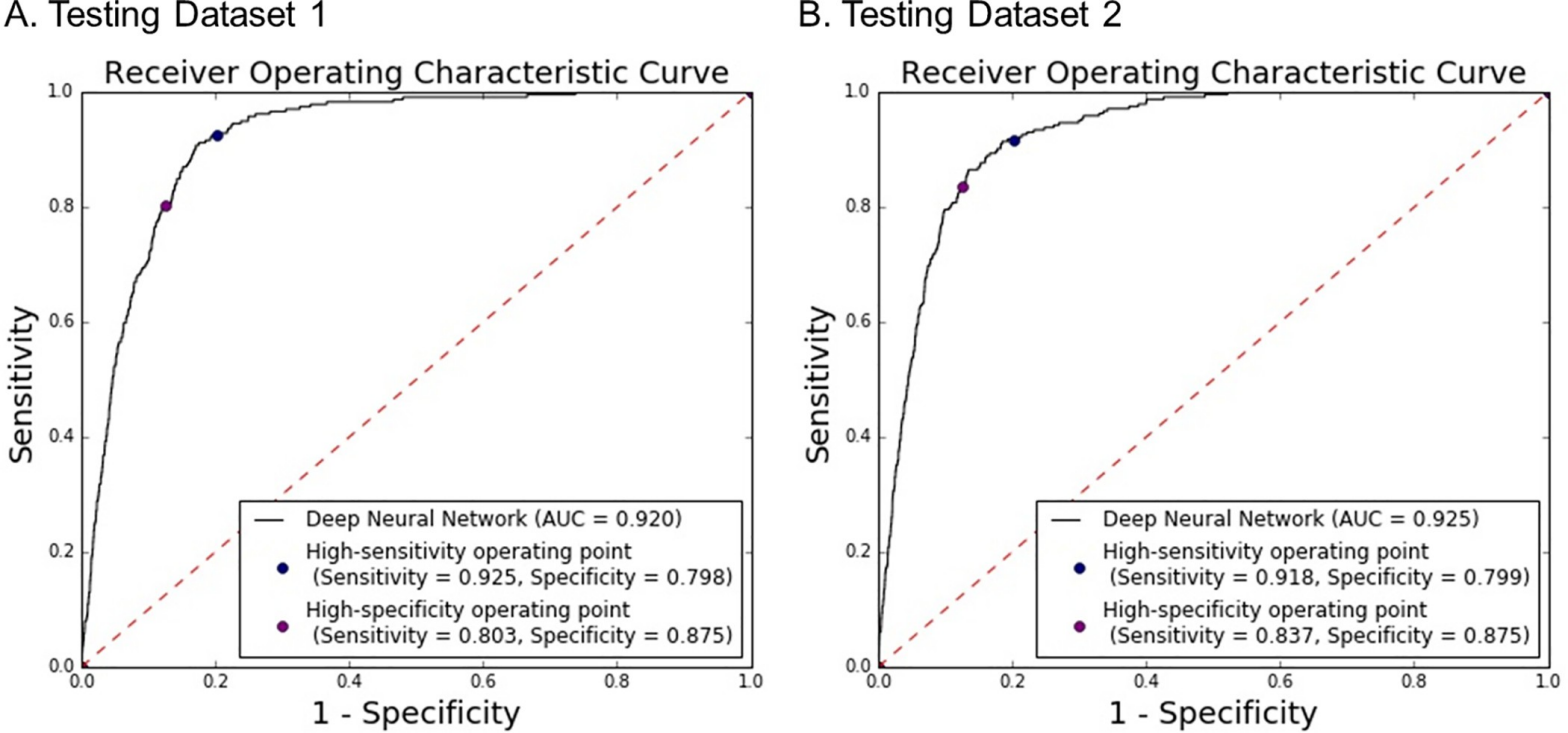
**Performance of the deep learning model for predicting 3 year stroke occurrence in (A) testing dataset 1 and (B) testing dataset 2**.

Fig 2 shows performance of the algorithm in predicting 3 year stroke occurrence. The trained DNN model achieved AUC values of 0.920 (95% CI, 0.908–0.932) and 0.925 (95% CI, 0.914–0.937) in testing datasets 1 and 2. Under the high sensitivity operating point (with cut point of calibrated model output probability 0.001), the sensitivity and specificity were 92.5% and 79.8% in testing dataset 1; 91.8% and 79.9% in testing dataset 2. Under the high specificity operating point (with cut point of calibrated probability 0.004), the algorithm obtained sensitivity and specificity of 80.3% and 87.5% in testing dataset 1; 83.7% and 87.5% in testing dataset 2. These findings corresponded to a negative predictive value of 99.97% for both testing dataset 1 and 2. The algorithm showed similar performance in both the male and the female population (S3 Fig). These results demonstrate that the DNN model can reliably estimate stroke risk using the health-related information in the EHR data. After applying Platt calibration to the DNN model outputs, the Hosmer-Lemeshow test (p-value for the original DNN model: <0.001, p-value for the model with Platt calibration: 0.039) and calibration curves showed an improvement of model calibration without altering AUC values (S4 Fig).

### Stroke rate in each risk category

As mentioned above, the DNN estimates stroke risk probability. We classified these continuous values into 5 risk categories. Fig 1 shows the 3 year and 8 year stroke rate of patients in each risk category in the designated testing datasets. The overall 3 year and 8 year stroke incidence rates of this population are 0.29% and 1.00%. When using the high sensitivity operating point (sensitivity 92.2%, specificity 79.9%), those who were classified as low risk (category 1, with calibrated probabilities 0–0.001) had a 3 year stroke rate of 0.03% and an 8 year stroke rate of 0.13%. When using the high specificity operating point (sensitivity 82.0%, specificity 87.5%), those who were classified as high risk (categories 3 to 5, with calibrated probabilities 0.004–0.013, 0.013–0.039, 0.039–0.066, respectively) had 3 year stroke rates of 0.93% to 3.55% and 8 year stroke rates of 3.96% to 9.60%. S3 Table showed the characteristics of patients in each risk category. Patients in higher risk categories were older and had a higher percentage of men, co-morbidities and medication use histories than those in lower risk categories (all with p<0.01). For assessing the diagnosis reliability, we tested the established model under different definitions of stroke events. Performance of the DNN did not change much after adjusting for stroke event definition (S1 Table).

### Sensitivity analyses

**Fig 3:**
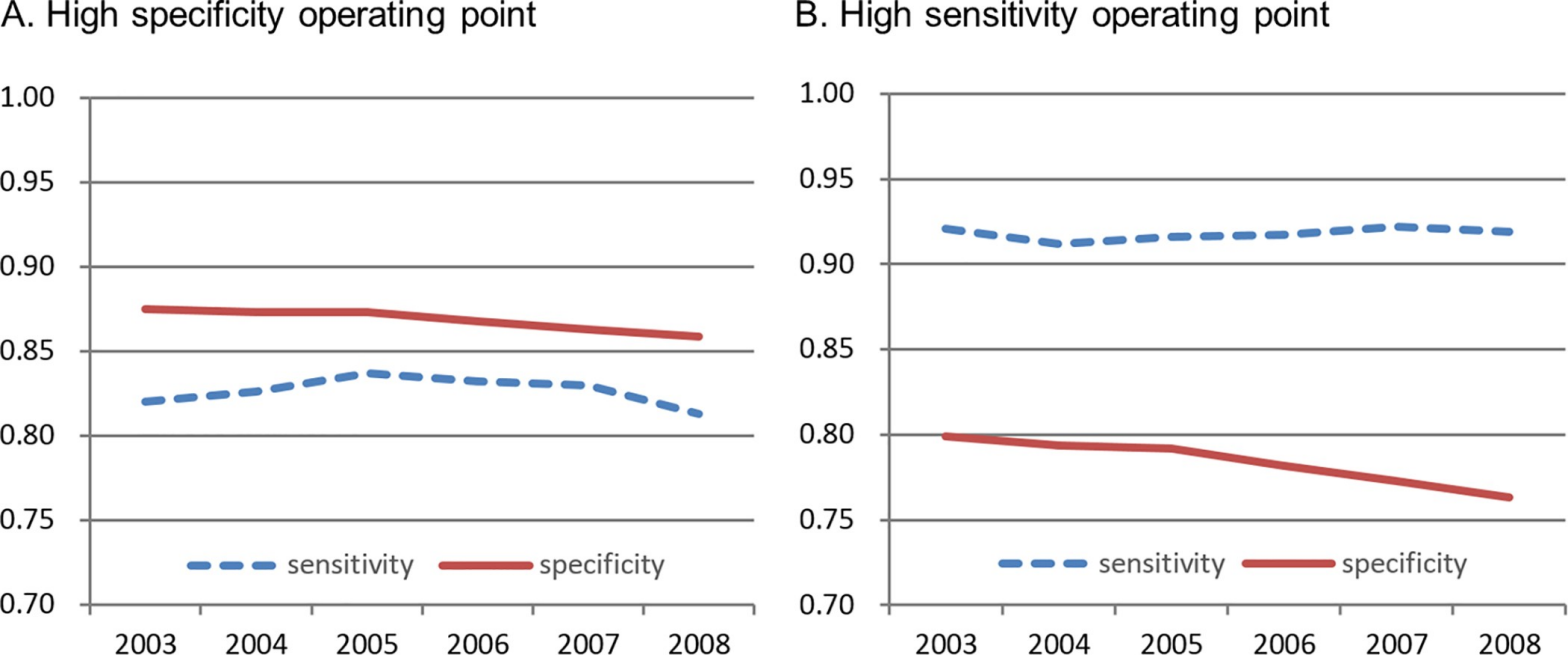
**Sensitivity and specificity of the DNN model for predicting 3 year stroke occurrence in different testing time periods under (A) the high specificity operating point and (B) the high sensitivity operating point**.

Additional sensitivity analyses were conducted in different testing time periods. Fig 3 and S5 Fig summarized performance of the 3 year stroke prediction algorithm (developed with 2003 data) when tested at different testing time periods (2003, 2004, 2005, 2006, 2007 and 2008). Performance of the model decreased only slightly (AUC values went from 0.923 to 0.909, specificity values went from 0.875 to 0.859 under the high specificity operating point, and sensitivity values went from 0.921 to 0.919 under the high sensitivity operating point). These results showed that the DNN model maintained high predictive ability 5 years after being developed. In another sub-sampling experiment, the effects on the quantity of development data upon algorithm performance were examined, i.e., predictive models were trained with varying numbers of records (S2 Fig). AUC values of these different models increased as we increased the development data amount, and plateaued after the data amount exceeded 3 million records (approximate 250,000 individuals).

### Comparing to other stroke risk assessment scores

Due to lack of some stroke risk factors in our database, we could only indirectly compare the performance of the DNN with other stroke risk assessment scores. Table 2 summarizes performance of currently available risk assessment scores and the established DNN model in different age ranges and gender subgroups. We compared the DNN model with other widely used stroke and cardiovascular risk assessment scores, including the Framingham,[4,49] QRISK1,[5] ASSIGN,[50] Reynolds,[51,52] QRISK2,[53] and QRISK3[54] scoring systems. Performance of these stroke prediction scores was retrieved from published papers.[4,5,49–54] The age ranges and gender characteristics of these testing populations are listed. We selected different testing populations from our testing datasets according to these gender and age characteristics to assess the DNN performance. As shown in the table, AUC values of the DNN model were higher than all of these existing clinical assessment scores. Performance of the DNN model is nearly identical to the most recently established QRISK3 scoring system. These results suggest that the DNN model derived from the claim database is quite competitive to those of currently available risk assessment tools.

**Table 2:** Performance of currently available stroke risk assessment scores and the deep learning model.

Characteristics of testing population ^a^	No. in corresponding population in our testing datasets	Performance of the DNN model, AUC (95% CI)	Performance of current scores, AUC, name of stroke risk score, published year	
age 35–74, women	39,248	0.870 (0.845–0.896)	0.774	Framingham, 1991 [4,5]
			0.788	QRISK1, 2007 [5]
			0.784	ASSIGN, 2007 [5,50]
			0.817	QRISK2, 2008 [53]
age 35–74, men	35,595	0.832 (0.803–0.862)	0.760	Framingham, 1991 [4,5]
			0.767	QRISK1, 2007 [5]
			0.764	ASSIGN, 2007 [5,50]
			0.792	QRISK2, 2008 [53]
age 30–74, women	46,450	0.887 (0.864–0.909)	0.774	Framingham, 2008 [49]
age 30–74, men	41,913	0.853 (0.826–0.879)	0.835	Framingham, 2008 [49]
age >45, women	27,458	0.819 (0.795–0.842)	0.809	Reynolds, 2007 [51]
age >50, men	19,629	0.746 (0.718–0.775)	0.708	Reynolds, 2008 [52]
age 25–84, women	56,929	0.896 (0.877–0.915)	0.880	QRISK3, 2017 [54]
age 25–84, men	51,796	0.870 (0.851–0.890)	0.858	QRISK3, 2017 [54]

^a^ Age ranges and gender characteristics of testing populations in reference papers.

AUC = area under the receiver operating characteristic curve; CI = confidence intervals.

## Discussion

### Main findings

To the best of our knowledge, this population-based cohort study is one of the largest studies for ischemic stroke prediction in an Asian population. Our results show that a DNN algorithm can reliably estimate future stroke risk in different age range and gender populations by using information from the EHR source. Meanwhile, the algorithm achieves comparable and sometimes better performance than current risk assessment tools. This high performing automated system maintains its stability across several years–strengthening the possibility of real world clinical adoption of this method.

### Automatic stroke risk assessment system

Stroke risk assessment is an important element in disease prevention.[3] Preventive interventions and frequent assessments are needed for those with high stroke risk to mitigate risks of detrimental events. Several stroke risk assessment scores have been developed for this purpose, such as the Framingham,[4,49] QRISK,[5] ASSIGN,[50,53,54] and Reynolds[51,52] scoring systems. Efforts to improve performance of these systems have been extensively studied in recent decades.[8] The most updated QRISK3[54] score adds several new stroke risk factors into the former QRISK2 model, including blood pressure variability, additional diseases and medications usage information. While the scoring system improves with these changes, the complexity of implementing such an assessment also increases. While the QRISK3 score can be easily used in the United Kingdom healthcare system, the implementation in other countries can still be difficult due to different designs in the recording of electronic health information. Moreover, the methods require additional time-consuming measurements beyond those used in routine clinical care (e.g., blood pressure variability and detailed family history).

Our DNN model, which estimates stroke risk by analyzing only the EHR, shows a competitive performance to the QRISK3 score and superior performance to other risk scores. While cloud-based health care systems, such as PharmaCloud[55], have been successfully implemented on the National Health Insurance system in Taiwan since 2015, physicians now have real-time access to patients' medication and disease diagnosis records in any clinic or hospital in Taiwan. In addition to avoiding duplicate prescriptions, we may use these EHRs for disease risk assessment by using this DNN model in the future. The rapid, unobtrusive, and automatic nature of this predictive model, just like other IDSSs (such as the Stroke Riskometer app[56]), can easily be applied to the existing healthcare systems. Physicians may therefore spend less time (within seconds) than other non-automatic tools (few minutes) for disease risk assessment in busy clinics. Meanwhile, the financial cost of such a DNN based IDSS may need further evaluation before clinical application.

### Validations of the deep learning model

The deep learning method has achieved breakthrough results across a variety of AI tasks in recent years.[32] Our recent work has shown that DNN can get better performance than simpler machine learning methodologies in analysis of this large-scale EHR. For predicting 5-year stroke occurrence, the DNN and gradient boosting decision tree approach can result in higher AUC values than the logistic regression and support vector machine approaches.[34] However, due to its complexity and unknown efficiency in clinical settings, further analyses are required to adopt its use as a clinical IDSS.[17] In this work, we validated the DNN model for different age ranges and gender populations (see Table 2 and S3 Fig). The model achieves higher AUC values than most risk scores and a competitive result to the most recent QRISK3 score. Additional issues may arise as clinical interventional strategies and patterns of medication use change over time. Our study demonstrates, however, that the model can perform well for up to 5 years after the development data. In this study, not only does the DNN model show high accuracy, but the clinical applicability is also validated.

### Study strengths

There are several strengths of this work. Herein, we demonstrate that DNN can be a promising method to perform disease prediction tasks. Using this novel data-driven approach to develop an automated stroke risk assessment system offers several benefits, e.g., rapid risk evaluations, no additional measurements beyond usual clinical practice, and high accuracy. This IDSS can automatically use EHR to estimate a patient’s relative stroke risk category within seconds, and may assist a physician’s clinical decision making for stroke preventive interventions, especially in a busy clinic. Furthermore, this predictive algorithm maintains flexibility in having multiple plausible operating points, such that the sensitivity and specificity can be adjusted to match the clinical requirements. For instance, the high sensitivity operating point can be used to identify those with very low stroke risk for avoiding unnecessary healthcare expense, and the high specificity point can be used for improving disease prevention for high risk patients. For another clinical need, different thresholds for these operating points could be chosen after a detailed cost-benefit analysis.[57] This single predictive model achieving high performance across a broad range of ages for both women and men is desirable in real world usage.

### Study limitations

There exist some limitations in this study. First, the study database comes from a medical claim data source. Several important known stroke risk factors, such as family history, cholesterol levels or smoking habits, are not explicitly recorded. Therefore, we are not able to directly compare the performance of the DNN on this population with the current clinical risk assessment scores, e.g. the Framingham and QRISK models. Although the AUC value is the most popular method for assessing risk prediction accuracy, several limitations still exist.[48] However, owing to the limited data in the research papers of other clinical scores, other methods (such as sensitivity, specificity, and reclassification table methods[58]) could hardly be applied for model comparison in this study. Second, although the predictive variables are generated from claim records, the present study does not account for dosing data of medications. Third, it is hard to perceive the relationships between variables explicitly.[15] It requires large amounts of computational power. Many existing analysis approaches and guidelines often use a linear model, which suffers from loss of predictive power. Some of the variables used in our model (such as health care cost and utilization) are not traditional stroke risk factors, and it remains ambiguous what kind of causal clinical variables available in EHRs, if any, we should consider when constructing models for predicting diseases. Therefore, this model may not directly provide preventive suggestions as the Framingham or other risk scores would, but it can serve as an easily and rapidly used IDSS. Fourth, although we used two testing datasets with no overlapping subjects, the validation process could be further strengthened in a future prospective study. We did not perform the cross validation process (a method for validating a machine learning model through generating different combinations of the data) in this study. Because several sub-sampling experiments in a variety of clinical scenarios were done in this study, applying the cross validation method would be a huge task and the results are not expected to change much. Fifth, the primary outcome in the current study was defined as any ischemic stroke in inpatient records. This may underestimate the occurrence of stroke due to failure to include patients who died out of hospital because of a very severe condition or those with less severe stroke who were treated in an outpatient clinic.

Sixth, while randomly under-sampling non-stroke data serves as an important method for managing the class imbalanced task, this method may make the output predictive probability much different from the observed disease risk (poor model calibration).[59] Although the Platt scaling method can improve the calibration of a machine learning model, the Hosmer-Lemeshow test (a statistical test that measures the differences between observed and predicted outcomes over the risk groups; if there is not a good agreement, it will show statistically significant difference) implied that the DNN model was still not well-calibrated (p-value 0.039) after applying this process. However, the large sample size of this study may let the Hosmer-Lemeshow test yield false-positive results.[48] On the other hand, although under-sampling produces poorly calibrated model probability, previous studies have shown that it provides better predictive discrimination (the ability of a disease predictive model to correctly assign a higher risk to a patient who is truly at a higher disease risk).[45,48,59] Applying the Platt scaling method improved the poorly calibrated output probability without altering the model discrimination (AUC value) in this study. Identifying these high risk patients still has clinical benefits since patients in risk category 5 would have higher risk than patients in other categories at 8 year follow-up. In clinical practical guidelines, patients with high (defined as a 10-year risk 5 to 10%, equivalent to risk category 3 and 4) and very high (a 10-year risk higher than 10%, equivalent to risk category 5) cardiovascular risk should receive pharmacological intervention for disease prevention.[60] Although we got a high negative predictive value, a high false positive rate was noted due to the fact that our model was applied on a sample representative of the general population and most people are at very low risk of stroke (positive predictive value at high specificity point was 1.85%, 6.4 times higher than randomly guessing [0.29%]). Therefore, this model may serve as a screening rather than a diagnostic IDSS. Finally, potential selection bias may happen in our study design. Those who did not have any medical contacts in the study period were not included. Furthermore, the study subjects included mainly Chinese and feasibility of this algorithm may be largely limited to this population due to some cultural or behavioral habits. Further study applying this method to other claims databases with different ethnic populations would be desired.

## Conclusions

In this study, our DNN model shows high performance in estimating future risk of ischemic stroke. Combining the use of DNN and EHR allows a rapid and potentially more precise stratification in identifying those patients with high stroke risk. Further prospective research is necessary to determine the feasibility of applying this algorithm in clinical practice and to see whether such a DNN based IDSS could improve stroke prevention in the general population.

## Supporting information

S1 Fig Performance of models (AUC values in testing datasets) developed with different numbers of features. (PDF)

S2 Fig Performance of models (AUC values in testing datasets) developed with different numbers of records. (PDF)

S3 Fig **Performance of the deep learning model for predicting 3 year stroke occurrence in (A) women and (B) men**.
(PDF)

S4 Fig **Calibration curves of the (A) deep learning model and (B) deep learning model with Platt calibration**.
(PDF)

S5 Fig Receiver operating characteristic curves of the deep learning model over time. (PDF)

S1 Table Model performance (AUC values) under different stroke event definitions in testing datasets 1 and 2. (PDF)

S2 Table The 300 features used for developing the deep learning model in this study. (PDF)

S3 Table Characteristics of patients in the 5 risk categories in the testing datasets. (PDF)
