# Supplementary material for: Development of an intelligent decision support system for ischemic stroke risk assessment in a population-based electronic health record database
Source: PLoS One. 2019 Mar 13;14(3):e0213007. doi: 10.1371/journal.pone.0213007 (PMC6415884; doi:10.1371/journal.pone.0213007)

S3 Fig. Performance of the deep learning model for predicting 3 year stroke occurrence in (A) women and (B) men.

A. Women

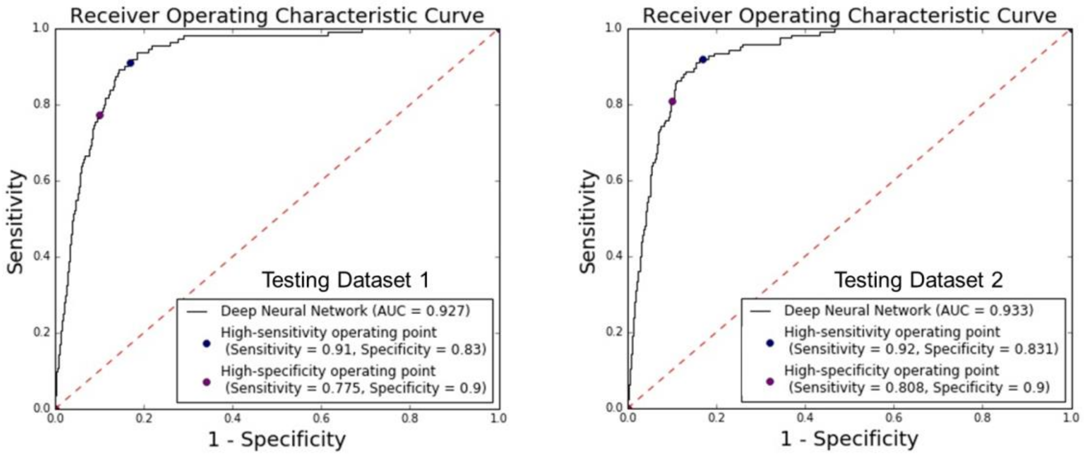

B. Men

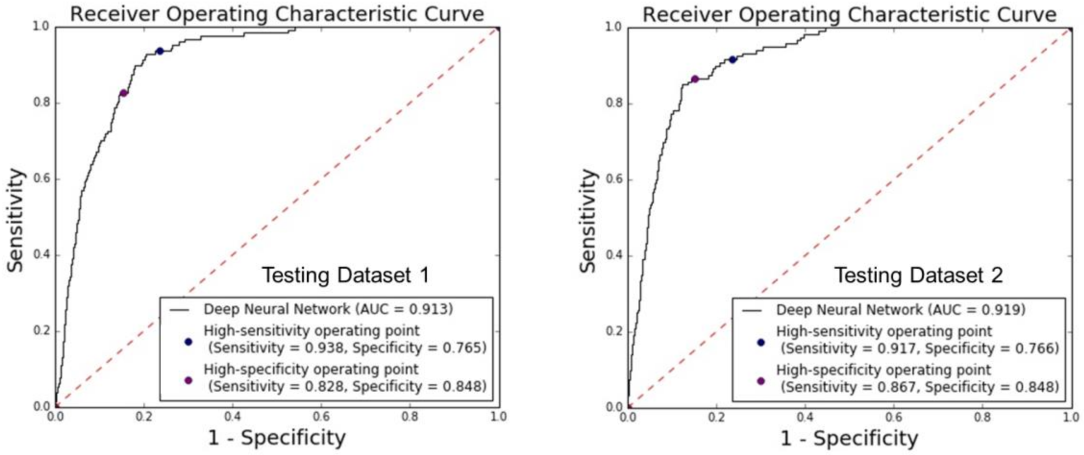

Supplement: S3 Fig — Performance of the deep learning model for predicting 3 year stroke occurrence in (A) women and (B) men. (PDF) [file pone.0213007.s003.pdf]
