# Supplementary material for: Development of an intelligent decision support system for ischemic stroke risk assessment in a population-based electronic health record database
Source: PLoS One. 2019 Mar 13;14(3):e0213007. doi: 10.1371/journal.pone.0213007 (PMC6415884; doi:10.1371/journal.pone.0213007)
Supplement: S4 Fig — Calibration curves of the (A) deep learning model and (B) deep learning model with Platt calibration. (PDF) [file pone.0213007.s004.pdf]

S4 Fig. Calibration curves of the (A) deep learning model and (B) deep learning model with Platt calibration.

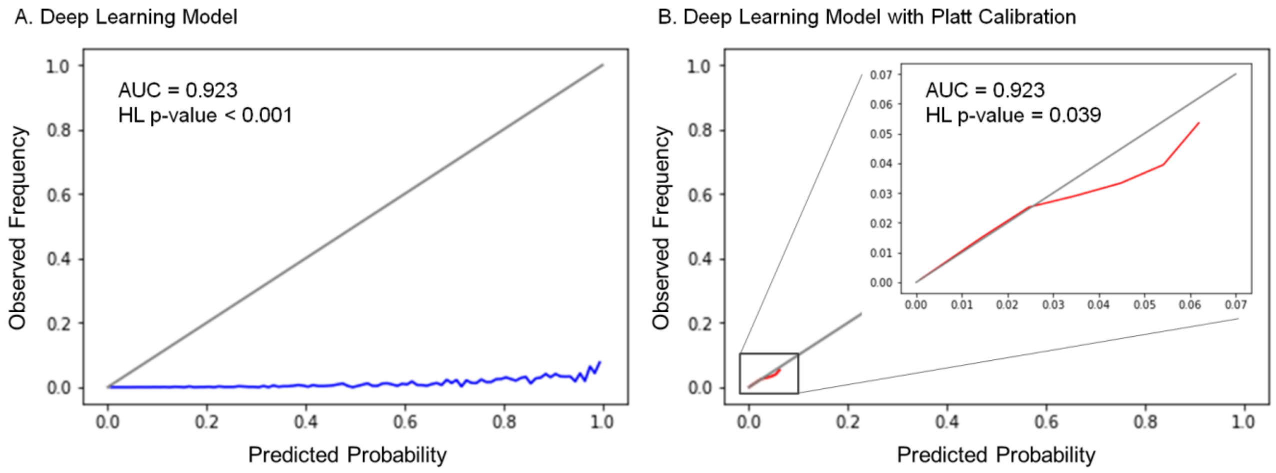

Model calibration was evaluated using calibration plots and the Hosmer-Lemeshow (HL) test. Observed frequency refers to the proportion of participants who were observed to have ischemic stroke. Predicted probability refers to the predicted probabilities generated by the models. AUC is the area under the receiver operating curve (C-statistic) for the models.
