# Supplementary figures and images for: Development of an intelligent decision support system for ischemic stroke risk assessment in a population-based electronic health record database
Source: PLoS One. 2019 Mar 13;14(3):e0213007. doi: 10.1371/journal.pone.0213007 (PMC6415884; doi:10.1371/journal.pone.0213007)

**S5 Fig. Receiver operating characteristic curves of the deep learning model over time.**

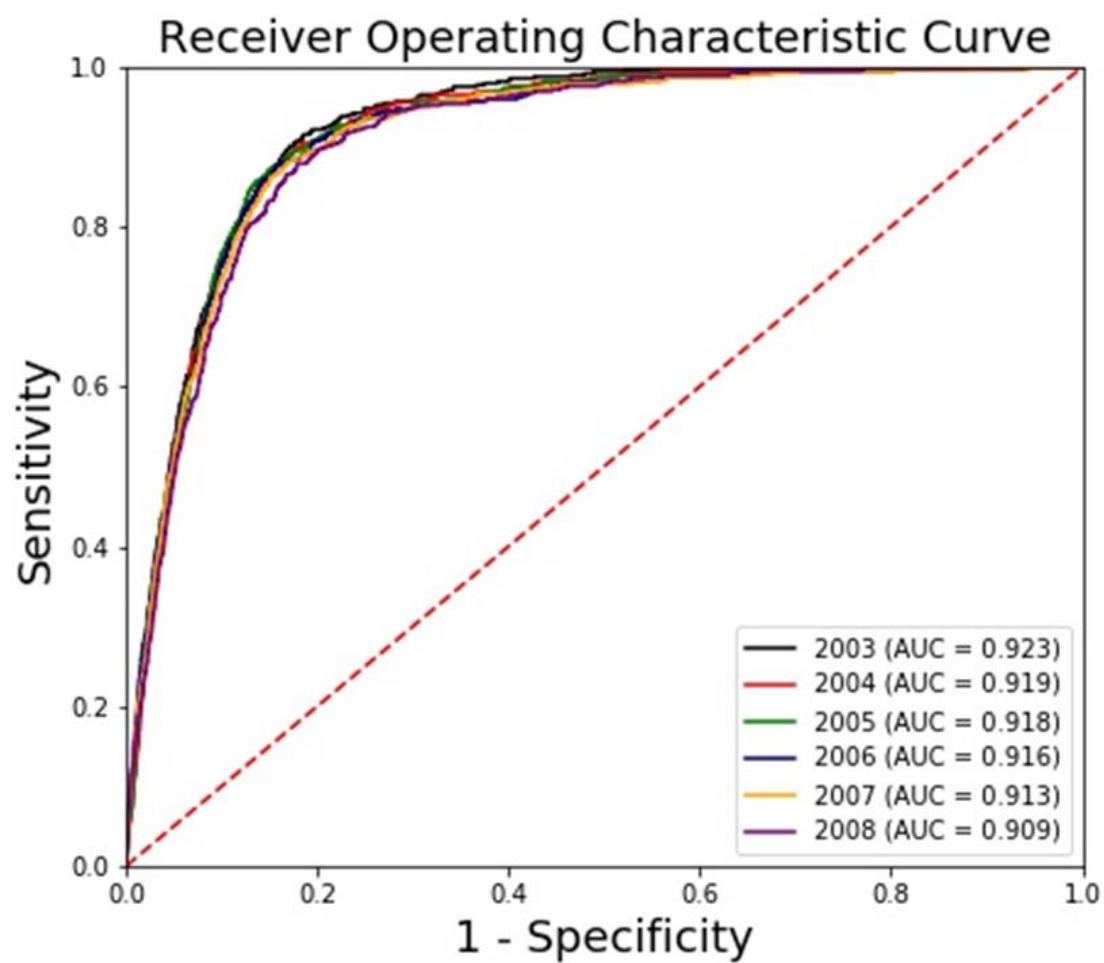

Supplement: S5 Fig — (PDF) [file pone.0213007.s005.pdf]
