# Supplementary material for: Development of an intelligent decision support system for ischemic stroke risk assessment in a population-based electronic health record database
Source: PLoS One. 2019 Mar 13;14(3):e0213007. doi: 10.1371/journal.pone.0213007 (PMC6415884; doi:10.1371/journal.pone.0213007)
Supplement: S1 Table — (PDF) [file pone.0213007.s006.pdf]

S1 Table. Model performance (AUC values) under different stroke event definitions in testing datasets 1 and 2.

Stroke event definitions	Testing dataset 1	Testing dataset 2
Stroke event defined as any diagnosis in inpatient records	0.920	0.925
Stroke event defined as principal or 2nd or 3rd diagnoses in inpatient records	0.919	0.926
Stroke event defined as only principal diagnosis in inpatient records	0.918	0.925
