# Supplementary material for: Development of an intelligent decision support system for ischemic stroke risk assessment in a population-based electronic health record database
Source: PLoS One. 2019 Mar 13;14(3):e0213007. doi: 10.1371/journal.pone.0213007 (PMC6415884; doi:10.1371/journal.pone.0213007)
Supplement: S2 Table — (PDF) [file pone.0213007.s007.pdf]

S2 Table. The 300 features used for developing the deep learning model in this study.

Categories	Descriptions of features
Demographics	Age
	Gender
Information of prescription and outpatient fee	Mean of prescription duration in the past 3 months
	Mean of prescription amount in the past 3 months
	Mean of prescription charge in the past 3 months
	Mean of pill count in the past 3 months
	SD of prescription duration in the past 3 months
	SD of prescription amount in the past 3 months
	SD of diagnostic fee in the past 3 months
	SD of prescription charge in the past 3 months
	SD of self-payment fee in the past 3 months
	SD of pill count in the past 3 months
	Mean of prescription duration in the past 6 months
	Mean of prescription amount in the past 6 months
	Mean of prescription charge in the past 6 months
	Mean of pill count in the past 6 months
	SD of prescription duration in the past 6 months
	SD of prescription amount in the past 6 months
	SD of diagnostic fee in the past 6 months
	SD of prescription charge in the past 6 months
	SD of outpatient cost in the past 6 months
	SD of self-payment fee in the past 6 months
	SD of insurance payment in the past 6 months
	SD of pill count in the past 6 months
	Mean of prescription duration in the past 1 year
	Mean of prescription amount in the past 1 year
	Mean of prescription charge in the past 1 year
	Mean of self-payment fee in the past 1 year
	Mean of pill count in the past 1 year
	SD of prescription duration in the past 1 year
	SD of prescription amount in the past 1 year
	SD of prescription charge in the past 1 year
	SD of outpatient cost in the past 1 year
	SD of self-payment fee in the past 1 year
	SD of insurance payment in the past 1 year
	SD of pill count in the past 1 year

	Mean of prescription duration in the past 2 years
	Mean of prescription amount in the past 2 years
	Mean of prescription charge in the past 2 years
	Mean of self-payment fee in the past 2 years
	Mean of pill count in the past 2 years
	SD of prescription duration in the past 2 years
	SD of prescription amount in the past 2 years
	SD of prescription charge in the past 2 years
	SD of outpatient cost in the past 2 years
	SD of self-payment fee in the past 2 years
	SD of insurance payment in the past 2 years
	SD of pill count in the past 2 years
	Mean of prescription duration in the past 3 years
	Mean of prescription amount in the past 3 years
	Mean of prescription charge in the past 3 years
	Mean of self-payment fee in the past 3 years
	Mean of pill count in the past 3 years
	SD of prescription duration in the past 3 years
	SD of prescription amount in the past 3 years
	SD of prescription charge in the past 3 years
	SD of outpatient cost in the past 3 years
	SD of self-payment fee in the past 3 years
	SD of insurance payment in the past 3 years
	SD of price per pill in the past 3 years
	SD of pill count in the past 3 years
Information of clinic management and specific treatment	No of outpatient clinic records in the past 3 months
	No of prescription records in the past 3 months
	No of operative dentistry records in the past 3 months
	No of periodontitis records in the past 3 months
	No of dental prosthesis records in the past 3 months
	No of pathology service records in the past 3 months
	No of dermatomyositis records in the past 3 months
	No of glaucoma records in the past 3 months
	No of trigeminal neuralgia records in the past 3 months
	No of nursing home records in the past 3 months
	No of smoking cessation records in the past 3 months
	No of HIV treatment records in the past 3 months
	No of outpatient clinic records in the past 6 months

	No of prescription records in the past 6 months
	No of operative dentistry records in the past 6 months
	No of periodontitis records in the past 6 months
	No of dental prosthesis records in the past 6 months
	No of pathology service records in the past 6 months
	No of intensive care records in the past 6 months
	No of CVS clinic records in the past 6 months
	No of dermatomyositis records in the past 6 months
	No of glaucoma records in the past 6 months
	No of trigeminal neuralgia records in the past 6 months
	No of nursing home records in the past 6 months
	No of smoking cessation records in the past 6 months
	No of HIV treatment records in the past 6 months
	No of outpatient clinic records in the past 1 year
	No of prescription records in the past 1 year
	No of operative dentistry records in the past 1 year
	No of periodontitis records in the past 1 year
	No of dental prosthesis records in the past 1 year
	No of OMFS clinic records in the past 1 year
	No of pathology service records in the past 1 year
	No of intensive care records in the past 1 year
	No of CVS clinic records in the past 1 year
	No of dermatomyositis records in the past 1 year
	No of glaucoma records in the past 1 year
	No of trigeminal neuralgia records in the past 1 year
	No of spinal injury records in the past 1 year
	No of MRI service records in the past 1 year
	No of nursing home records in the past 1 year
	No of smoking cessation records in the past 1 year
	No of HIV treatment records in the past 1 year
	No of outpatient clinic records in the past 2 years
	No of prescription records in the past 2 years
	No of operative dentistry records in the past 2 years
	No of periodontitis records in the past 2 years
	No of dental prosthesis records in the past 2 years
	No of OMFS clinic records in the past 2 years
	No of pathology service records in the past 2 years
	No of intensive care records in the past 2 years

	No of CVS clinic records in the past 2 years
	No of peptic ulcer records in the past 2 years
	No of dermatomyositis records in the past 2 years
	No of glaucoma records in the past 2 years
	No of vitreous hemorrhage records in the past 2 years
	No of corneal degeneration records in the past 2 years
	No of purpura records in the past 2 years
	No of trigeminal neuralgia records in the past 2 years
	No of spinal injury records in the past 2 years
	No of seborrheic dermatitis records in the past 2 years
	No of MRI service records in the past 2 years
	No of nursing home records in the past 2 years
	No of smoking cessation records in the past 2 years
	No of HIV treatment records in the past 2 years
	No of outpatient clinic records in the past 3 years
	No of prescription records in the past 3 years
	No of operative dentistry records in the past 3 years
	No of periodontitis records in the past 3 years
	No of dental prosthesis records in the past 3 years
	No of OMFS clinic records in the past 3 years
	No of pathology service records in the past 3 years
	No of intensive care records in the past 3 years
	No of CVS clinic records in the past 3 years
	No of peptic ulcer records in the past 3 years
	No of dermatomyositis records in the past 3 years
	No of glaucoma records in the past 3 years
	No of vitreous hemorrhage records in the past 3 years
	No of corneal degeneration records in the past 3 years
	No of anemia records in the past 3 years
	No of purpura records in the past 3 years
	No of trigeminal neuralgia records in the past 3 years
	No of spinal injury records in the past 3 years
	No of seborrheic dermatitis records in the past 3 years
	No of MRI service records in the past 3 years
	No of prescription refill records in the past 3 years
	No of nursing home records in the past 3 years
	No of smoking cessation records in the past 3 years
	No of HIV treatment records in the past 3 years

Information of disease (ICD-10-CM code)	No of E11 diagnosis records in the past 3 months
	No of I10 diagnosis records in the past 3 months
	No of R42 diagnosis records in the past 3 months
	No of Z23 diagnosis records in the past 3 months
	No of E11 diagnosis records in the past 6 months
	No of I10 diagnosis records in the past 6 months
	No of I11 diagnosis records in the past 6 months
	No of R42 diagnosis records in the past 6 months
	No of Z23 diagnosis records in the past 6 months
	No of E11 diagnosis records in the past 1 year
	No of H10 diagnosis records in the past 1 year
	No of H25 diagnosis records in the past 1 year
	No of I10 diagnosis records in the past 1 year
	No of I11 diagnosis records in the past 1 year
	No of J01 diagnosis records in the past 1 year
	No of M54 diagnosis records in the past 1 year
	No of R42 diagnosis records in the past 1 year
	No of Z00 diagnosis records in the past 1 year
	No of Z23 diagnosis records in the past 1 year
	No of E11 diagnosis records in the past 2 years
	No of H10 diagnosis records in the past 2 years
	No of H25 diagnosis records in the past 2 years
	No of I10 diagnosis records in the past 2 years
	No of I11 diagnosis records in the past 2 years
	No of I25 diagnosis records in the past 2 years
	No of J01 diagnosis records in the past 2 years
	No of K02 diagnosis records in the past 2 years
	No of M54 diagnosis records in the past 2 years
	No of R42 diagnosis records in the past 2 years
	No of Z00 diagnosis records in the past 2 years
	No of Z23 diagnosis records in the past 2 years
	No of E11 diagnosis records in the past 3 years
	No of E78 diagnosis records in the past 3 years
	No of H25 diagnosis records in the past 3 years
	No of I10 diagnosis records in the past 3 years
	No of I11 diagnosis records in the past 3 years
	No of I25 diagnosis records in the past 3 years
	No of J01 diagnosis records in the past 3 years

	No of K02 diagnosis records in the past 3 years
	No of M54 diagnosis records in the past 3 years
	No of R42 diagnosis records in the past 3 years
	No of Z00 diagnosis records in the past 3 years
	No of Z23 diagnosis records in the past 3 years
Information of medication (ATC code)	No of A02AA use records in the past 3 months
	No of A02AF use records in the past 3 months
	No of A06AB use records in the past 3 months
	No of A10BA use records in the past 3 months
	No of A10BB use records in the past 3 months
	No of B01AC use records in the past 3 months
	No of C01DA use records in the past 3 months
	No of C08CA use records in the past 3 months
	No of C09AA use records in the past 3 months
	No of C09CA use records in the past 3 months
	No of N05BA use records in the past 3 months
	No of N07CA use records in the past 3 months
	No of A02AA use records in the past 6 months
	No of A02AF use records in the past 6 months
	No of A06AB use records in the past 6 months
	No of A10BA use records in the past 6 months
	No of A10BB use records in the past 6 months
	No of B01AC use records in the past 6 months
	No of C01DA use records in the past 6 months
	No of C03CA use records in the past 6 months
	No of C07AB use records in the past 6 months
	No of C08CA use records in the past 6 months
	No of C08DB use records in the past 6 months
	No of C09AA use records in the past 6 months
	No of C09CA use records in the past 6 months
	No of N05BA use records in the past 6 months
	No of N07CA use records in the past 6 months
	No of A02AA use records in the past 1 year
	No of A02AF use records in the past 1 year
	No of A06AB use records in the past 1 year
	No of A10BA use records in the past 1 year
	No of A10BB use records in the past 1 year
	No of B01AC use records in the past 1 year

	No of C01DA use records in the past 1 year
	No of C03CA use records in the past 1 year
	No of C07AB use records in the past 1 year
	No of C08CA use records in the past 1 year
	No of C08DB use records in the past 1 year
	No of C09AA use records in the past 1 year
	No of C09CA use records in the past 1 year
	No of C10AA use records in the past 1 year
	No of G04CA use records in the past 1 year
	No of J07BB use records in the past 1 year
	No of M01AB use records in the past 1 year
	No of M03BB use records in the past 1 year
	No of N02BE use records in the past 1 year
	No of N05BA use records in the past 1 year
	No of N07CA use records in the past 1 year
	No of S01AB use records in the past 1 year
	No of A02AA use records in the past 2 years
	No of A02AF use records in the past 2 years
	No of A02AX use records in the past 2 years
	No of A06AB use records in the past 2 years
	No of A10BA use records in the past 2 years
	No of A10BB use records in the past 2 years
	No of B01AC use records in the past 2 years
	No of B05XA use records in the past 2 years
	No of C01DA use records in the past 2 years
	No of C03CA use records in the past 2 years
	No of C04AC use records in the past 2 years
	No of C07AB use records in the past 2 years
	No of C08CA use records in the past 2 years
	No of C08DB use records in the past 2 years
	No of C09AA use records in the past 2 years
	No of C09CA use records in the past 2 years
	No of C10AA use records in the past 2 years
	No of G04CA use records in the past 2 years
	No of J07BB use records in the past 2 years
	No of M01AB use records in the past 2 years
	No of M01AH use records in the past 2 years
	No of M03BB use records in the past 2 years

	No of N02BE use records in the past 2 years
	No of N05BA use records in the past 2 years
	No of N07CA use records in the past 2 years
	No of S01AB use records in the past 2 years
	No of S01XA use records in the past 2 years
	No of A02AA use records in the past 3 years
	No of A02AF use records in the past 3 years
	No of A02AG use records in the past 3 years
	No of A02AX use records in the past 3 years
	No of A06AB use records in the past 3 years
	No of A10BA use records in the past 3 years
	No of A10BB use records in the past 3 years
	No of A11EA use records in the past 3 years
	No of B01AC use records in the past 3 years
	No of B05XA use records in the past 3 years
	No of C01DA use records in the past 3 years
	No of C03CA use records in the past 3 years
	No of C04AC use records in the past 3 years
	No of C04AD use records in the past 3 years
	No of C07AB use records in the past 3 years
	No of C08CA use records in the past 3 years
	No of C08DB use records in the past 3 years
	No of C09AA use records in the past 3 years
	No of C09CA use records in the past 3 years
	No of C10AA use records in the past 3 years
	No of G04CA use records in the past 3 years
	No of J07BB use records in the past 3 years
	No of M01AB use records in the past 3 years
	No of M01AC use records in the past 3 years
	No of M01AH use records in the past 3 years
	No of M03BB use records in the past 3 years
	No of N02BE use records in the past 3 years
	No of N05BA use records in the past 3 years
	No of N07CA use records in the past 3 years
	No of S01AB use records in the past 3 years
	No of S01XA use records in the past 3 years

SD = standard deviation; HIV = human immunodeficiency virus; CVS = cardiovascular surgery; OMFS = Oral and maxillofacial surgery; MRI = magnetic resonance imaging
