# Supplementary material for: Development of an intelligent decision support system for ischemic stroke risk assessment in a population-based electronic health record database
Source: PLoS One. 2019 Mar 13;14(3):e0213007. doi: 10.1371/journal.pone.0213007 (PMC6415884; doi:10.1371/journal.pone.0213007)
Supplement: S3 Table — (PDF) [file pone.0213007.s008.pdf]

S3 Table. Characteristics of patients in the 5 risk categories in the testing datasets.

Characteristics	Category 1	Category 2	Category 3	Category 4	Category 5
No. of patients	134,048	12,796	9,455	9,100	2,874
DNN estimated probabilities	0-0.3	0.3-0.5	0.5-0.7	0.7-0.9	0.9-1
Probabilities after Platt calibration	0-0.001	0.001-0.004	0.004-0.013	0.013-0.039	0.039-0.066
No. of patients with stroke events within 3 years, No. (%) [absolute risk]	38 (0.03) [0.0003]	49 (0.38) [0.0038]	88 (0.93) [0.0093]	207 (2.27) [0.0227]	102 (3.55) [0.0355]
No. of patients with stroke events within 8 years, No. (%) [absolute risk]	174 (0.13) [0.0013]	221 (1.73) [0.0173]	374 (3.96) [0.0396]	643 (7.07) [0.0707]	276 (9.60) [0.0960]
No. of OPD visits in 2003, median (IQR)	9 (4-17)	15 (7-25)	18 (10-30)	24 (14-37)	32 (20-48)
Men, No. (%)	62,559 (46.7)	6,796 (53.1)	5,281 (55.9)	5,514 (60.6)	1,844 (64.2)
Age in years, mean (SD)	28.0 (14.4)	56.4 (6.9)	64.5 (7.7)	72.4 (8.5)	78.8 (8.1)
Co-morbidity, No. (%)					
Hypertension	4,030 (3.0)	3,665 (28.6)	4,279 (45.3)	5,705 (62.7)	2,233 (77.7)
Hyperlipidemia	4,581 (3.4)	2,549 (19.9)	2,319 (24.5)	2,446 (26.9)	788 (27.4)
Diabetes mellitus	2,216 (1.7)	1,576 (12.3)	1,816 (19.2)	2,795 (30.7)	1,394 (48.5)
Ischemic heart disease	1,693 (1.3)	1,176 (9.2)	1,544 (16.3)	2,426 (26.7)	1,109 (38.6)
Atrial fibrillation	45 (0.0)	45 (0.4)	76 (0.8)	168 (1.8)	125 (4.3)
Heart failure	206 (0.2)	169 (1.3)	367 (3.9)	772 (8.5)	495 (17.2)
Medication use, No. (%)					
Antiplatelet agents	7,159 (5.3)	2,194 (17.1)	2,842 (30.1)	4,450 (48.9)	2,091 (72.8)
Renin angiotensin system inhibitors	1,902 (1.4)	2,059 (16.1)	2,654 (28.1)	4,175 (45.9)	1,954 (68.0)
Beta blockers	9,435 (7.0)	3,473 (27.1)	3,406 (36.0)	4,172 (45.8)	1,499 (52.2)
Calcium channel blockers	3,192 (2.4)	2,852 (22.3)	3,491 (36.9)	5,011 (55.1)	2,160 (75.2)
Other antihypertensive drugs	5,101 (3.8)	2,708 (21.2)	3,357 (35.5)	4,682 (51.5)	2,019 (70.3)
Statins	798 (0.6)	907 (7.1)	1,054 (11.1)	1,402 (15.4)	533 (18.5)
Oral hypoglycemic agents	963 (0.7)	987 (7.7)	1,386 (14.7)	2,392 (26.3)	1,328 (46.2)
Insulins	132 (0.1)	115 (0.9)	213 (2.3)	436 (4.8)	333 (11.6)

DNN = deep neural network; OPD = outpatient department; IQR = interquartile range; SD = standard deviation.
